# Surgical treatment of eyelid sequelae due to trachoma performed by a few São Paulo hospitals of the Brazilian Unified Health System

**DOI:** 10.5935/0004-2749.2024-0279

**Published:** 2025-04-07

**Authors:** Vera Helena Turola Machado Joseph, Norma Helen Medina, Maria de Fátima Costa Lopes, Juliana Albano de Guimarães, Antonio Augusto Velasco e Cruz

**Affiliations:** 1 Centro de Oftalmologia Sanitária, Centro de Vigilância Epidemiológica, Secretaria de Estado da Saúde, São Paulo, SP, Brazil; 2 Coordenação Geral de Doenças em Eliminação, Departamento de Doenças Transmissíveis, Secretaria de Vigilância em Saúde e Ambiente, Ministério da Saúde, Brasília, DF, Brazil; 3 Departamento de Oftalmologia, Otorrinolaringologia e Cirurgia de Cabeça e Pescoço, Faculdade de Medicina de Ribeirão Preto, Universidade de São Paulo, Ribeirão Preto, SP, Brazil

**Keywords:** Trachoma, Trichiasis, Medical records, Epidemiology, Neglected diseases, Unified Health System, Brazil

## Abstract

**Purpose:**

Trachoma is the major infectious cause of preventable blindness in the world, and its sequelae include the presence of cicatricial entropion and trachomatous trichiasis. Trachoma can be corrected by surgical treatment of the eyelids and, if left untreated, may result in corneal opacification, low vision, and blindness. There are limited data on trachomatous trichiasis in Brazil. This study was conducted to estimate the frequency of entropion and trichiasis surgeries of trachomatous origin based on the records of procedures performed in specialized hospitals that served the Unified Health System (SUS) in the years 2016 and 2017.

**Methods:**

This was a retrospective study conducted in the oculoplastic sectors of the ophthalmology services of the following three hospitals in the state of São Paulo: *Hospital das Clínicas da Faculdade de Medicina de Botucatu* (HC Botucatu), *Hospital das Clínicas da Faculdade de Medicina de Ribeirão Preto da Universidade de São Paulo* (HC Ribeirão Preto), and *Hospital Estadual de Bauru* (HE Bauru). Medical records corresponding to the codes of interest were evaluated.

**Results:**

In total, 462 medical records were evaluated, including 170 (36.8%) at HC Botucatu, 61 (13.2%) at HE Bauru, and 231 (50.0%) at HC Ribeirão Preto. There were 39 (8.4%) cases of trachomatous trichiasis, ranging from 9 (14.8%) at HE Bauru to 15 (6.5%) at HC Ribeirão Preto.

**Conclusions:**

The frequency of surgery due to trachoma was low in these oculoplastic services. The state of São Paulo might have reached the goal for trachoma elimination in the surgical component. The questionnaire used for data collection was successfully tested despite some difficulties in collecting data from the medical records. Studies with the same methodology are recommended in other services in the areas of endemic trachoma in the past to understand the frequency of eye lid surgeries performed for treating trachomatous sequelae.

## INTRODUCTION

Trachoma is the major infectious cause of preventable blindness, occurring most frequently in areas of low socioeconomic level. The World Health Organization (WHO) estimates that 103 million people live in endemic areas that require trachoma control actions, and trachoma is responsible for blindness and visual impairment in 1.9 million people^(1^,^2)^. In Brazil, the disease was endemic in the past century, and its prevalence started declining in the state of São Paulo only in the 1950s^(3^,^4)^. People who have had trachoma in their childhood may have had sequelae^(5^,^6)^.

The severity of trachoma directly correlates with the frequency of reinfection episodes. Trachoma is also associated with bacterial conjunctivitis of other etiologies, which facilitates transmission and increase the inflammatory reaction. This, in turn, leads to more intense conjunctival necrosis and scarring^(5^,^6)^.

Trachoma sequelae are generally found in individuals aged >15 years and are caused by repeated episodes of active trachoma that can lead to scarring of the upper eyelid conjunctiva and cicatricial entropion, resulting in trichiasis, with or without entropion. Trichiasis is an extremely painful condition and can be corrected by surgical treatment of the eyelids; however, if left untreated, it can, in combination with other ocular changes induced by trachoma, result in corneal opacification, low vision and blindness^(5^,^7)^.

According to the WHO, trachomatous trichiasis (TT) is defined as the presence of at least one eyelash on the upper eyelid touching the surface of the eyeball or the evidence of recent removal of inturned eyelashes from the upper eyelid, associated with the presence of scars on the superior tarsal conjunctiva (TS) suggestive of trachoma^(8^,^9)^. This definition excludes trichiasis that affects only the lower eyelid^(10)^.

The SAFE strategy, proposed by the WHO, consists of four interventions, including “S” for surgery for TT, “A” for antibiotics for ocular *Chlamydia trachomatis* infection, “F” for facial cleanliness, and “E” for environmental improvement^(11)^.

In 1998, the World Health Assembly adopted the resolution WHA 51.11 for targeting the elimination of blinding trachoma in the world and proposed that member countries actively implement the SAFE strategy^(12)^. In 2003, the WHO developed goals for eliminating trachoma and subsequently reformulated them to validate the elimination of trachoma as a public health problem. These standard operating procedures include the following technical elimination goals^(13^,^14)^:

• Prevalence of trachomatous inflammation-follicular (TF) of <5% in children aged 1-9 years, sustained by at least two years of absence, after the mass administration of antibiotics, in each previously endemic district;
• Prevalence of TT unknown to the health system, of <0.2% in individuals aged ≥15 years (where cases unknown to the health system exclude individuals with postoperative trachomatous trichiasis, individuals who declined surgery and individuals who have not yet been operated on but for whom surgery has already been scheduled) in each previously endemic district;
• Written evidence of the health system’s commitment to identifying and managing incident cases of TT using defined strategies and evidence of appropriate financial resources to implement these strategies.

There is limited information on TT in Brazil, where trachoma is not a notifiable condition in all states. Therefore, there is a lack of methods to understand the real magnitude of TT cases to confirm that we have reached the elimination goal in the surgical (S) component.

The records of surgical procedures, including those related to trichiasis, were obtained from the Department of Informatics of the Unified Health System (DATASUS) in the databases of the Outpatient Information System of the Unified Health System - SIA/SUS and in the Hospital Information System - SIH/SUS. These databases present limitations for registering procedures related to the treatment of trichiasis and surgical correction of entropion, because they do not have specific fields to report cases of trachomatous entropion and TT^(15)^. Therefore, data on TT in Brazil present restrictions for evaluating the disease status in recent years; therefore, we decided to analyze the information found in the medical records of some hospitals that performed eyelid surgeries.

Brazil registered in the SUS/Data base from January 2015 to April 2018, 2956 reports related to “the surgical treatment of trichiasis with or without graft” and 2819 reports related to “the surgical correction of entropion and ectropium”. In the SIA/SUS for the procedure of “Surgical treatment of trichiasis with or without graft, 39.3% of the surgeries were performed in hospitals of the São Paulo State and 18% were executed in hospitals of Paraná state. The procedure “Surgical correction of entropion and ectropion 45.4% were done in São Paulo state and 10% in Rio Grande do Sul. Regarding the SIH/SUS, 39% were in São Paulo State and 10.2% in Paraná state^(15)^. These data justify our choice to investigate hospitals in the state of São Paulo. The selected hospitals were some of those with a larger number of surgeries performed in those years registered in the SUS database.

The purpose of this study was to review the medical records of patients who underwent procedures related to entropion/trichiasis in 2016 and 2017 to identify patients with a trachomatous etiology.

## METHODS

This was a retrospective study conducted in the oculoplastic sectors of the ophthalmology services of three hospitals in the state of São Paulo, with approval obtained from the Ethics Committee of Hospital das Clínicas da Faculdade de Medicina de Ribeirão Preto da Universidade de São Paulo (HC Ribeirão Preto).

The three hospitals in São Paulo were *Hospital das Clínicas da Faculdade de Medicina de Botucatu* (HC Botucatu), *Hospital das Clínicas da Faculdade de Medicina de Ribeirão Preto da Universidade de São Paulo* (HC Ribeirão Preto), and *Hospital Estadual de Bauru* (HE Bauru).

Initially, the SIA/SUS and SIH/SUS databases of DATASUS were consulted for the procedure codes of interest. Because the databases do not specify the procedures of trachomatous origin, all medical records corresponding to the codes of interest in 2016 and 2017 were evaluated.

There are specific codes for each procedure, including those related to trichiasis (of any etiology) as follows: “Surgical treatment of trichiasis with or without graft”, code 0405010192; “Surgical correction of entropion and ectropion”, code 0405010010; and “Epilation of eyelashes”, code 0405010060.

Three healthcare professionals analyzed and recorded the information found in the medical records. TT was considered only when there was trichiasis or entropion of the upper eyelid, trachomatous scarring on the tarsal conjunctiva (TS), Herbert pits, upper pannus, or history of trachoma^(16)^. Exclusion criteria were cases of ectropion irrespective of the eyelid, trichiasis of other etiologies (such as blepharitis, meibomitis, ocular pemphigoid, Stevens-Johnson syndrome, burns, trauma, tumors, herpes zoster, leprosy, and other cicatricial anomalies of the eyelid margin, associated or not with previous surgeries), and trichiasis of the lower eyelid only.

A questionnaire developed by the oculoplastic experts of the Ministry of Health was applied to collect data and evaluate the feasibility, applicability, and reliability of the instrument.

## RESULTS

Data collection performed in the databases between 2016 and 2017 in Brazil identified 801 procedures of “surgical treatment of trichiasis with or without graft” in 55 healthcare institutions, 3198 procedures of “surgical correction of entropion and ectropion” in 85 institutions, and 1114 procedures of “epilation of eyelashes” in 71 institutions.

A total of 480 procedures were performed in 2016 and 2017 in the oculoplastic sectors of the ophthalmology services of the selected hospitals in the state of São Paulo (Table 1).

**Table 1:** Number of procedures identified in the SIA/SUS and SIH/SUS databases with their codes, by institutions where they were performed

Procedure	HE Bauru		HC Botucatu		HC Ribeirão Preto		Total	
	nº	%	nº	%	nº	%	nº	%
Surgical treatment of trichiasis with or without graft- 0405010192	17	24.3	33	19.5	83	34.4	133	27.7
Surgical correction of entropion and ectropion- 0405010010	34	48.6	134	79.3	111	46.1	279	58.1
Epilation of eyelashes- 0405010060	19	27.1	2	1.2	47	19.5	68	14.2
TOTAL	70	100.0	169	100.0	241	100.0	480	100.0

Source: DATASUS SIA/SUS and SIH/SUS

We evaluated 462 (96.3%) medical records, including 170 (36.8%) at HC Botucatu (electronic medical records), 61 (13.2%) at HE Bauru (electronic medical records), and 231 (50.0%) at HC Ribeirão Preto (paper medical records). The same patient may have undergone more than one procedure (Table 2).

**Table 2:** Number of medical records analyzed and number of people with trachomatous trichiasis (TT) by municipality and gender

Municipality	Number of medical records analyzed	Number of people with trachomatous trichiasis				Total	
		Male		Female		nº	%
	nº	nº	%	nº	%		
Bauru	61	6	9.8	3	4.9	9	14.8
Botucatu	170	10	5.9	5	2.9	15	8.8
Ribeirão Preto	231	4	1.7	11	4.8	15	6.5
Total	462	20	4.3	19	4.1	39	8.4

Source: HE Bauru, HC Botucatu, and HC Ribeirão Preto.

Of the 462 medical records analyzed, trachomatous etiology was identified in 39 cases (8.4%). Patients’ ages ranged from 49 to 93 years, with a mean age of 75 years. Regarding gender, there were 51.3% men and 48.7% women. All patients resided in the interior of the state of São Paulo, and 59% of them were born in the state of São Paulo. At least 35% of them had recurrence of trichiasis after surgery.

## DISCUSSION

Trachoma sequelae, generally found in individuals aged >15 years, are characterized by scarring of the upper eyelid conjunctiva and cicatricial entropion (inward-turning eyelids) with or without trichiasis, where eyelashes touch the eye.

In this study, 8.4% of patients had cicatricial entropion and TT secondary to trachoma sequelae, which is a relatively low incidence considering the high prevalence of trachoma in the state of São Paulo in the past^(4)^.

These findings suggest that the state of São Paulo has reached the goal for trachoma elimination in the surgical component (S), with a few cases of TT and has a health system organized capable of identifying and managing the incident cases of TT.

The limitations of this study include the inherent challenges of analyzing retrospective data. The major difficulties encountered in this study were the technical medical jargon that is difficult to be interpreted by non-ophthalmologist researchers, the lack of complete information in the records, the incorrect completion of surgical codes, and the time spent analyzing each medical record to identify the trachomatous etiology. Regarding the first difficulty, we would suggest the presence of an ophthalmologist during the analysis of medical records. Moreover, it would be useful to have more specific surgical codes. Therefore, the Ministry of Health proposed the registration of procedures with the specification of trachomatous etiology, including two International Classification of Diseases codes in the SIA/SUS and SIH/SUS databases, the primary disease code-B94.0-sequelae of trachoma and the secondary code H02.0- entropion and trichiasis of the eyelid^(15)^. Despite the inherent difficulties, this study achieved the objective of revealing the frequency of entropion/trichiasis surgeries due to trachomatous etiology.

We believe that this type of study would be helpful if conducted in other surgical ophthalmological services to build a profile of TT surgeries in Brazil.

We also hope that this study can highlight the importance of the correct completion of surgical records regarding cicatricial entropion and trichiasis due to trachoma. Thereby, it will be feasible to collaborate with data to be included in the dossier for WHO to validate the elimination of trachoma as a public health problem.
